# *In Vitro* Phosphorylation Does not Influence the Aggregation Kinetics of WT α-Synuclein in Contrast to Its Phosphorylation Mutants

**DOI:** 10.3390/ijms15011040

**Published:** 2014-01-15

**Authors:** Sarah Schreurs, Melanie Gerard, Rita Derua, Etienne Waelkens, Jean-Marc Taymans, Veerle Baekelandt, Yves Engelborghs

**Affiliations:** 1 Laboratory of Biomolecular Dynamics, KU Leuven, Celestijnenlaan 200G, Leuven B-3001, Belgium; E-Mail: schreurs.sarah@gmail.com; 2 Laboratory of Biochemistry, KU Leuven Kulak, Etienne Sabbelaan 53, Kortrijk B-8500, Belgium; E-Mail: melanie.gerard@kuleuven-kulak.be; 3 Laboratory of Protein Phosphorylation and Proteomics, KU Leuven, O&N I Herestraat 49-bus 901, Leuven B-3000, Belgium; E-Mails: rita.derua@med.kuleuven.be (R.D.); etienne.waelkens@med.kuleuven.be (E.W.); 4 Laboratory for Neurobiology and Gene Therapy, KU Leuven, Kapucijnenvoer 33, Leuven B-3000, Belgium

**Keywords:** alpha-synuclein, phosphorylation, parkinson’s disease, phosphorylation mutants, FKBP, *in vitro* fibrillization

## Abstract

The aggregation of alpha-synuclein (α-SYN) into fibrils is characteristic for several neurodegenerative diseases, including Parkinson’s disease (PD). Ninety percent of α-SYN deposited in Lewy Bodies, a pathological hallmark of PD, is phosphorylated on serine129. α-SYN can also be phosphorylated on tyrosine125, which is believed to regulate the membrane binding capacity and thus possibly its normal function. A better understanding of the effect of phosphorylation on the aggregation of α-SYN might shed light on its role in the pathogenesis of PD. In this study we compare the aggregation properties of WT α-SYN with the phospho-dead and phospho-mimic mutants S129A, S129D, Y125F and Y125E and *in vitro* phosphorylated α-SYN using turbidity, thioflavin T and circular dichroism measurements as well as transmission electron microscopy. We show that the mutants S129A and S129D behave similarly compared to wild type (WT) α-SYN, while the mutants Y125F and Y125E fibrillate significantly slower, although all mutants form fibrillar structures similar to the WT protein. In contrast, *in vitro* phosphorylation of α-SYN on either S129 or Y125 does not significantly affect the fibrillization kinetics. Moreover, FK506 binding proteins (FKBPs), enzymes with peptidyl-prolyl *cis-trans* isomerase activity, still accelerate the aggregation of phosphorylated α-SYN *in vitro*, as was shown previously for WT α-SYN. In conclusion, our results illustrate that phosphorylation mutants can display different aggregation properties compared to the more biologically relevant phosphorylated form of α-SYN.

## 1. Introduction

The aggregation of alpha-synuclein (α-SYN) into fibrils is characteristic for several neurodegenerative diseases, including Parkinson’s disease (PD) [1]. These fibrils are deposited into Lewy bodies (LB) and Lewy neurites, the pathological hallmarks of PD [2]. α-SYN is a 140 amino acids long intrinsically disordered protein with almost no secondary structural elements and only limited tertiary intramolecular contacts under physiological conditions *in vitro* [3,4]. Although the exact function of α-SYN remains elusive, it might play a role in regulating dopamine neurotransmission [5,6], vesicular trafficking [7,8] and modulating synaptic function and plasticity [9–11]. The majority (~90%) of the α-SYN protein in LBs is phosphorylated at S129, compared to only about 4% in normal brain extracts [12–14]. However, it remains unresolved whether phosphorylation occurs before or after aggregation. It is also still unclear whether this modification promotes or inhibits aggregation and what effect it has on the neurotoxicity *in vivo*. It was shown that the tertiary intramolecular long-range interactions are disrupted in *in vitro* phosphorylated α-SYN, which might cause the inhibition of aggregation observed by Paleologou *et al.* [15]. Contradictory results were obtained by Fujiwara *et al.* where *in vitro* phosphorylated α-SYN was more prone to aggregate [13].

Previous studies have used the phosphorylation mutants, S129A and S129D, to mimic absence of phosphorylation or constitutive phosphorylation respectively, in order to investigate this effect in cells and *in vivo*. While the phosphorylation mimicking mutant (S129D) promoted cell death in a Drosophila model [16] as well as in neuroblastoma cell lines [11,17], this was not the case in viral vector-mediated rat models. Whereas two studies report accelerated dopaminergic neuronal loss with the S129A mutant and a moderate to significant protective effect of the S129D mutant compared to wild-type α-SYN [18,19], McFarland and colleagues found no significant differences between both phosphorylation mutants and wild-type α-SYN in nigrostriatal toxicity or aggregation [20].

Several kinases have been shown to phosphorylate α-SYN *in vitro* on S129, such as casein kinase I (CKI), CKII [13,21,22], G-protein coupled receptor kinases (GRK) [23,24] and members of the polo-like kinase family (PLK2) [25,26]. CKI and CKII phosphorylate on S87 as well, albeit to a lesser extent [15,21]. Two recent studies showed that PLK2, also known as serum-inducible kinase (SNK), is a major contributor of pS129-α-SYN in primary neuronal cultures and in transgenic mice [25,26]. Moreover, PLK2 levels are elevated in brains of Alzheimer disease and dementia with LB patients [26].

*In vitro* phosphorylation on tyrosines of α-SYN has been described for several kinases such as Syk kinase [27], SRC kinases [28] and Fyn kinase, the latter specifically phosphorylating Y125 of α-SYN [29]. The phosphorylation on Y125 is thought to regulate α-SYN membrane binding and the inhibition of phospholipase D2 by α-SYN [30]. Phosphorylation on all three tyrosines in the *C*-terminal part by SYK kinase was reported to prevent aggregation *in vitro* [27]. Recently it was shown that tyrosine phosphorylation of α-SYN might protect against neurotoxicity in a Drosophila model of PD [31]. Overexpression of the Drosophila homologue of SYK kinase even rescued the toxicity of the phospho-mimicking mutant S129D.

In this study we performed a systematic comparison of the biochemical properties of normal α-SYN WT with α-SYN phosphorylation mutants (S129A/D and Y125F/E) and *in vitro* phosphorylated α-SYN. Our group previously showed that the enzyme FKBP12 (FK506 binding protein 12) (EC. 5.2.1.8.), which is a *cis*-*trans* proline isomerase from the family of FK506 binding proteins, accelerates the aggregation of WT α-SYN [32–34]. Since α-SYN’s five proline residues, as well as the major phosphorylation sites, are all located in the *C*-terminus, we investigated the influence of α-SYN phosphorylation at either S129 or Y125 on this acceleration.

Our results show that phosphorylation mutants of α-SYN do not fully reproduce the behaviour of α-SYN phosphorylated with physiologically relevant kinases. They also suggest that phosphorylation does not necessarily promote aggregation. Furthermore, the ability of FKBP12 to accelerate α-SYN aggregation is comparable between phosphorylated and WT α-SYN.

## 2. Results and Discussion

### 2.1. Construction and Production of the α-SYN Phosphorylation Mutants S129A/D and Y125F/E

**Figure 1.**
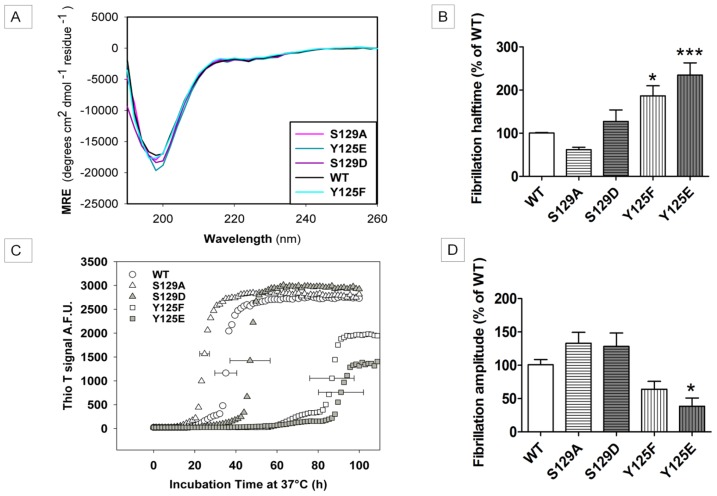

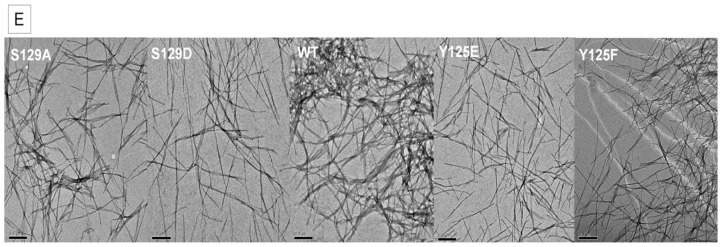
Far UV-CD spectra of WT α-SYN and its phosphorylation mutants and fibrillization of WT α-SYN compared to the phosphorylation mutants. (**A**) Far UV-CD spectra of α-SYN and phosphorylation mutants were taken at the start point of further measurements to account for possible secondary structure differences due to the mutations. All spectra have a minimum near 200 nm, typical for a random coil structure. The phosphorylation mutants (Y125F (cyan), Y125E (dark cyan), S129A (pink), S129D (purple)) show spectra similar to that of the WT protein (black), and our obtained values of approximately −20,000 MRE (mean residue ellipticity) correspond well to values of monomeric WT α-SYN found in literature. The kinetics of the fibrillization process of α-SYN WT and phospho-mutants were followed by a Thioflavin T assay under continuous shaking (270 rpm) at 37 °C. A concentration of 50 μM α-SYN was used in each experiment; (**B**) Mean values of the halftimes; The halftimes of fibrillization are expressed as percentages of WT, which was set to 100%; (**C**) Representative figure showing an inhibitory effect with both tyrosine mutants (Y125F (white squares) and Y125E (gray squares)). The kinetics of both serine mutants, however, are similar to that of the WT protein (S129A (white triangles), S129D (gray triangles) and WT (white circles)); (**D**) Mean end phase fluorescence intensities; (**B**) and (**D**) are calculated from 5 independent measurements (*n* = 5) each done in quadruplicate, with the standard error of mean (SEM) shown on each bar. * and *** indicate a statistical significant difference when compared to WT with a *p*-value < 0.05 and < 0.001 respectively; TEM images were taken and are represented in (**E**), from left to right: S129A, S129D, WT, Y125E, Y125F. The scale bars are set to 200 nm.

We wanted to generate phosphorylation mimicking mutants S129D and Y125E as well as their respective negative control mutants, S129A and Y125F in order to characterize their *in vitro* aggregation properties. By replacing Ser or Tyr with an acidic amino acid (Asp or Glu) one can mimic the electrostatic features of a phosphorylated residue, although it should be noted that the phosphate group on Ser or Tyr is fully deprotonated at pH 7.4 which results in a net charge of −2 instead of −1 with Asp/Glu. The structural similarities between Ser and Asp make this replacement a reasonable phospho-mimicking mutation, although it should be noted that the distance between the alpha carbon and the negative charge is longer in the phosphoserine compared to Asp. The structures of Tyr and Glu are much less alike, but since no aromatic acidic amino acid is available the choices are limited. Using far UV-CD measurements we could confirm that the spectra of all mutants were comparable to that of the WT protein and represented a predominantly random coil structure (Figure 1A), corresponding well with the values of monomeric WT α-SYN reported in literature [35].

### 2.2. *In Vitro* Aggregation Properties of the Phosphorylation Mutants

First, we assessed fibril formation by a thioflavin T (ThioT) assay (Figure 1B–D). The curves were fitted with a sigmoid model to obtain the half-time of aggregation (*t*_1/2(aggregation)_) as well as the end phase amplitude, which correlates to the amount of fibrils formed (Figure 1B,D). The fibrillization of both tyrosine mutants Y125E and Y125F was significantly slower with a half time approximately twice as long as for WT α-SYN (Figure 1B). Moreover, the plateau was reached at lower fluorescence intensities (Figure 1D). None of the serine mutants showed a significant difference when compared to WT α-SYN, although the S129A mutant tended to aggregate faster than the wild-type protein (Figure 1B,C). When the stationary phase was reached, electron microscopy analysis was used to examine the morphology of the aggregates formed (Figure 1E). Fibrils were present in all samples and their morphology was similar to that of WT α-SYN, confirming that differences in aggregation kinetics between α-SYN variants are due to true kinetic differences, rather than to hypothetical differences in aggregation pathways. Turbidity experiments were also performed to investigate the general aggregation properties (Supplementary Figure S1). Overall, the turbidity measurements of the phosphorylation mutants followed the same trends as the ThioT measurements. Due to a higher variation between independent measurements, the only significant difference in *t*_1/2(aggregation)_ was obtained between WT and the Y125F mutant (Supplementary Figure S1B), while the Y125E phosphorylation mimic mutant reached a significantly lower end phase amplitude (Supplementary Figure S1C).

### 2.3. *In Vitro* Phosphorylation of α-SYN on Different Residues

**Figure 2.**
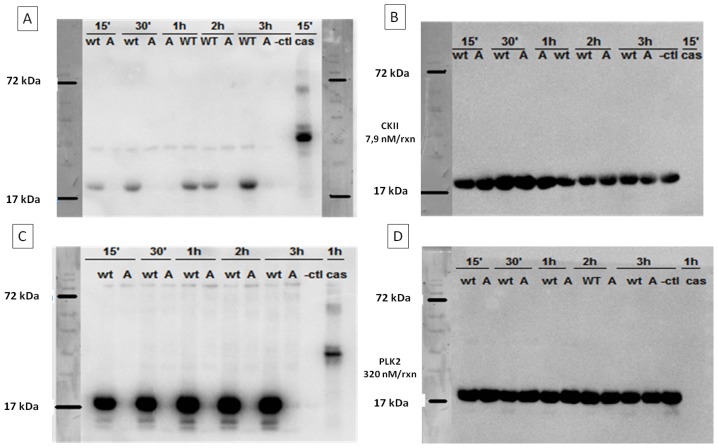

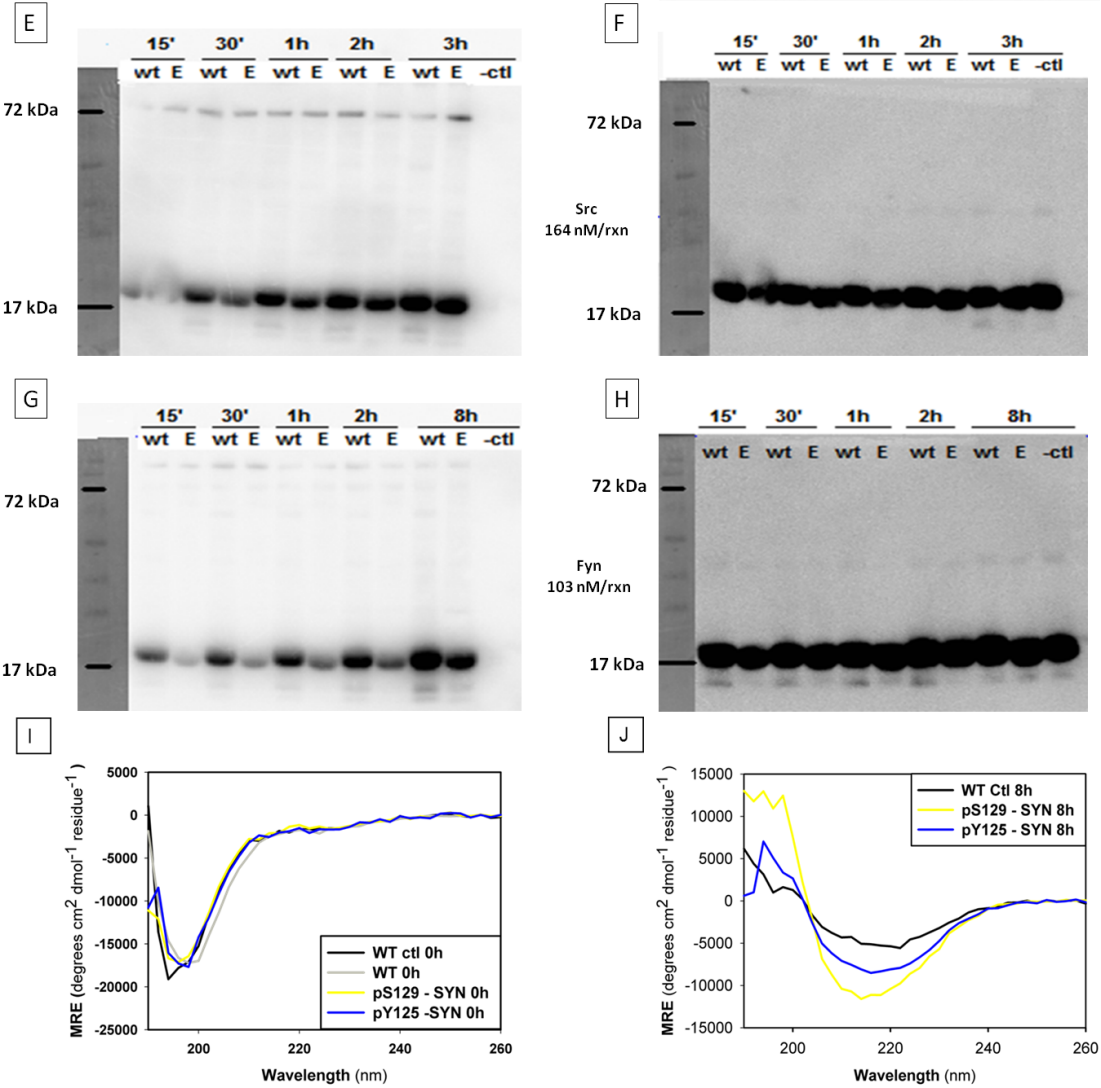
Phosphate incorporation using autoradiography and far UV-CD spectra of (phosphorylated)-α-SYN. Phosphate incorporation was monitored by a radioactivity assay, where ATP-P^32^ was incubated in the presence of different kinases and WT α-SYN or the S129A/ Y125E mutant. The reaction was stopped at different time points (15 min, 30 min, 1 h, 2 h, 3 h as well as 24 h for PLK2 and Fyn) with SDS-loading dye and boiling for 5 min. Radioactive phosphate incorporation was visualised by autoradiography, after which western blotting was performed to detect relative protein levels. For each time point WT and a respective mutant were used. WT: WT α-SYN, A: S129A α-SYN, E: Y125E α-SYN, -ctl: negative control (kinase boiled for five min prior to test). Cas: casein kinase, used as a positive control for serine phosphorylation. Kinases and the kinase concentrations used in the *in vitro* phosphorylation assays are given between the blot panels (please refer to the Materials and Methods section for full details on the kinases and phosphorylation procedure) (**A**) radio-activity blot of CKII phosphorylation; (**B**) western blot of (**A**); (**C**) radioactivity blot of PLK2 phosphorylation; (**D**) western blot of (**C**); (**E**) radioactivity blot of SRC phosphorylation; (**F**) western blot of (**E**); (**G**) radioactivity blot of Fyn phosphorylation; (**H**) western blot of (**G**); (**I**) Far UV-CD spectra of proteins after overnight phosphorylation. All spectra show a minimum near 200 nm comparable to that of WT α-SYN (black line), typical for a random coil structure. The manipulations necessary for phosphorylation and removal of ATP afterwards do not disturb the secondary structure (compare spectrum of WT control (black line) to that of the WT protein (gray line)). pY125-α-SYN: WT α-SYN phosphorylated by Fyn kinase (blue line) and pS129-α-SYN: WT α-SYN phosphorylated by PLK2 kinase (yellow line); and (**J**) Far UV–CD spectra of pS129-α-SYN (yellow line), pY125-α-SYN (cyan) and their control (WT ctl (black line)), prepared as in (**I**), after 8 h of continuous agitation. All samples show predominantly a β-sheet structure.

The reported specificity of Casein Kinase II (CKII) for phosphorylating α-SYN on S129 and to a lesser extent S87 [13,21,22] was first confirmed by *in vitro* phosphorylation of WT and S129A α-SYN using a radioactive assay. The phosphate incorporation was measured by autoradiography. Phosphorylation was indeed mainly on S129, since only after longer incubation times a band became visible with the S129A mutant (Figure 2A,B). PLK2 however, was even more specific in phosphorylating α-SYN on S129, including after 24 h incubation (Figure 2C,D) as reported before [26]. Using a scintillation counting based quantification of phosphate incorporation, we determined that PLK2 phosphorylates α-SYN very efficiently (0.72 phosphates per α-SYN molecule) unlike CKII phosphorylation where the yield was only about 2%. Therefore, we used PLK2 to phosphorylate α-SYN on S129 for all further experiments.

In view of reported differential effects of phosphorylation on Y125 compared to phosphorylation on S129 on cell death and α-SYN aggregation in a Drosophila model [31], we determined the influence of Y125 phosphorylation on the aggregation of α-SYN *in vitro*. We tested two kinases: SRC and Fyn kinase. SRC kinase did not phosphorylate specifically on Y125, as was shown by autoradiography (Figure 2E,F). Phosphorylation with Fyn kinase showed higher specificity for Y125 at early time points, although background signal in the Y125E control could be observed at longer incubation times indicating that a small proportion of other tyrosine sites are phosphorylated (Figure 2G,H). By calculation of the molar ratios of incorporated phosphates per α-SYN molecule, we determined that 1.49 phosphates per α-SYN molecule are incorporated for SRC phosphorylation and 1.18 for Fyn phosphorylation. Taking into account that the incorporation of a radioactive phosphoryl group in the Y125E mutants was 0.69 and 0.42 respectively, both kinases show a similar efficiency of Y125 phosphorylation (0.79 and 0.76 respectively). Since phosphorylation with Fyn kinase was more specific we decided to further use only this kinase.

### 2.4. Structural Properties of *in Vitro* Phosphorylated α-SYN

CD measurements were performed after the α-SYN phosphorylation (overnight phosphorylation, as described in materials and methods) to see if the incorporation of a phosphoryl group affects the native structure of the protein. All spectra correlated to an unfolded structure similar to the WT protein (Figure 2I).

For the control, unphosphorylated WT α-SYN, all manipulations of the phosphorylation protocol with the exception of ATP/kinase addition were carried out for all further experiments, to rule out any influence this assay might have on the aggregation properties of the protein. This control (indicated as “WT ctrl”) is also represented by an unfolded CD spectrum (Figure 2I).

Early oligomerisation events of α-SYN can be followed by FCS by the addition of trace amounts of A140C-α-SYN labeled with Alexa 488 [36]. With this technique a distribution of diffusion coefficients (*D*) is obtained, which correlates to the size of the diffusing particles. At the starting point of each experiment we observed a homogeneous distribution and the obtained *D* corresponds to the monomeric form of the protein [36]. This was also the case for α-SYN phosphorylated on S129 (pS129-α-SYN) as well as on Y125 (pY125-α-SYN) (Supplementary Figure S2). The mean diffusion coefficient of 4 independent measurements was calculated, which was similar for pS129-α-SYN (92.0 +/− 6.57 μm^2^/s), pY125-α-SYN (92.3 +/− 3.84 μm^2^/s) and their control (unphosphorylated WT α-SYN (WT ctl: 86.4 +/− 4.95 μm^2^/s)) (Supplementary Figure S2A). From the diffusion coefficients the hydrodynamic radius can be calculated, assuming a spherical particle, which ranged from 23.9 (+/− 1.4) Å for pY125-α-SYN, 24.3 (+/− 1.7) Å for pS129-α-SYN to 25.8 (+/− 2.9) Å for WT ctl (Supplementary Figure S2B). These values were not significantly different and correspond to the previously reported hydrodynamic radius for monomeric WT α-SYN of 26.6 Å [37] or 28.2 Å [15,38]. It should be noted that alternative techniques, such as NMR based techniques have yielded higher hydrodynamic radii for phosphorylated α-SYN (35.3 Å for S129 phosphorylated α-SYN) [15], perhaps because these techniques do not require assumptions on particle shape.

### 2.5. The Influence of pS129- and pY125-α-SYN on Its *in Vitro* Fibril Formation

**Figure 3.**
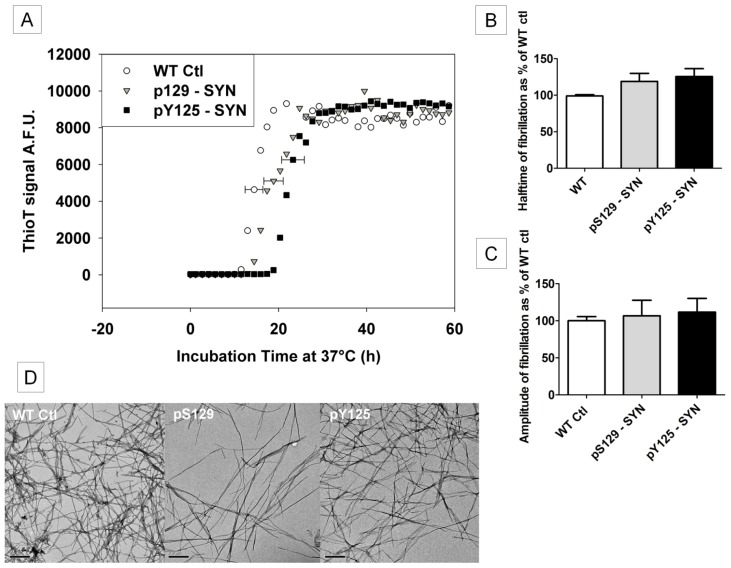
Fibrillization of (phosphorylated)-α-SYN. The kinetics of fibrillization of phosphorylated α-SYN (30 μM) monitored by ThioT fluorescence. Before the ThioT assay all samples were incubated overnight at 30 °C with ATP and the respective kinases. The next day ATP was removed by buffer exchange as described in Materials and Methods. The control was subjected to the same incubations/buffer exchange without the addition of ATP/kinase. (**A**) Representative measurement showing comparable kinetics of pY125-α-SYN (black squares), pS129-α-SYN (gray triangles) and WT ctl (white circles); (**B**) Mean halftimes and (**C**) mean end phase amplitudes, obtained from five independent measurements (*n* = 5) each done in quadruplicate, SEM is shown on each bar. The values of pS129-α-SYN and pY125-α-SYN are expressed as percent of their control (WT Ctl); and (**D**) TEM images were taken at the end of the measurements to confirm fibril formation. From left to right: WT ctl, pS129: pS129-α-SYN, pY125: pY125-α-SYN. Scale bars are set to 200 nm.

Next, we used the ThioT assay to assess fibril formation upon *in vitro* phosphorylation of α-SYN on either pS129 or Y125. A slightly longer *t*_1/2(aggregation)_ was observed compared to the unphosphorylated control (WT ctl), although this difference was not significant (Figure 3A,B). Moreover, no significant difference was observed in end phase amplitude (Figure 3A,C). These ThioT measurements show that *in vitro* phosphorylation of α-SYN does not significantly influence the fibrillization kinetics of α-SYN under the conditions tested. TEM images taken after the ThioT assay showed that both pS129- and pY125-α-SYN formed fibrils comparable to WT Ctl (Figure 3D), confirming that the phosphorylation also did not induce alternate forms of aggregation.

Please note that since we observed that the presence of ATP influences the aggregation of α-SYN (Supplementary Figure S3), ATP was removed by buffer exchange before the aggregation properties were determined.

Part of the samples used for the ThioT measurements was subjected to mass spectrometry to verify the phosphorylation state of the protein. With PLK2 only S129 was phosphorylated, and the extent of phosphorylation was determined to be 46% by MALDI-TOF/TOF analysis and 35% by LC-ESI-MS/MS analysis (See Supplementary Figure S4A). FYN kinase phosphorylated Y125, although Y133 or Y136 were also phosphorylated. The percentage of tyrosine phosphorylated alpha-synuclein was determined by MALDI-TOF/TOF analysis to be 71% and by LC-ESI-MS/MS analysis to be 52% (See Supplementary Figure S4B).

Next, CD measurements were carried out to follow the formation of β-sheet structure. pS129-α-SYN first started to form β-sheets after five h under continuous agitation. After eight h all samples showed mostly a β-sheet structure (Figure 2J). This correlates with the fibrillization kinetics seen in the ThioT measurements.

### 2.6. Does FKBP12 Still Accelerate the Fibrillization of Phosphorylated α-SYN?

**Figure 4.**
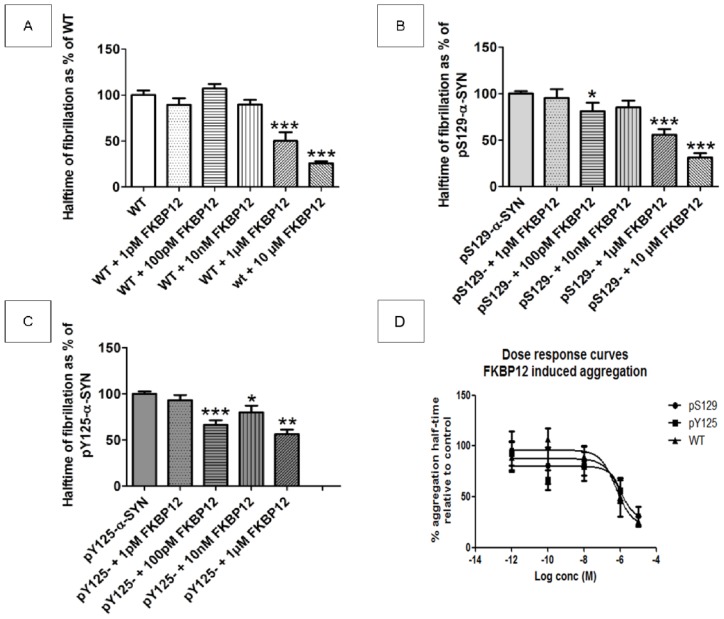
The influence of FKBP12 on the fibrillization of (phosphorylated)-α-SYN. The influence of FKBP12 on the fibrillization kinetics of WT α-SYN (**A**); pS129-α-SYN (**B**) and pY125-α-SYN (**C**) (all 30 μM). For FKBP12 a concentration range was used: no FKBP12, 1 pM FKBP12, 100 pM FKBP12, 10 nM FKBP12, 1 μM FKBP12 and 10 μM FKBP12. Mean halftimes are shown and were obtained from four independent measurements (*n* = 4) each done in quadruplicate, SEM is shown on the bars. The conditions with FKBP12 present are expressed as percentages of the respective control: α-SYN (WT, pS129 or pY125) without FKBP12, *, ** and *** indicate a statistical significant difference when compared to WT with a *p*-value < 0.05, a *p*-value < 0.01 or a *p*-value < 0.001 respectively; and (**D**) Values of aggregation half-times from Figure 4 are plotted in an *x*–*y* plot and a dose response curve is fitted using GraphPad Prism (least squares method, fit equation is *Y* = Bottom + (Top − Bottom)/(1 + 10^(^*^X^* ^– log^ *^EC^*^50)^). The *EC*_50_ values of FKBP12 in reducing the aggregation half-time of the different α-SYN variants are comparable for all experimental groups, ranging from 0.6 to 1 μM. WT, normal α-SYN; pS129, α-SYN phosphorylated at S129 by PLK2; pY125, α-SYN phosphorylated at Y125 by Fyn kinase.

It was previously shown in our lab that the enzyme FKBP12, which is a peptidyl–prolyl isomerase, accelerates the fibrillization of α-SYN [32–34,39]. Because in α-SYN all the proline residues are situated in the *C*-terminus (P108, P117, P120, P128, P138) [40], where also the phosphorylation events take place, we wondered whether phosphorylating the C-terminal part would affect this acceleration. It appears from the ThioT measurements that FKBP12 can still accelerate the fibrillization process of α-SYN phosphorylated on either residue (S129 or Y125). In our assay the acceleration of WT α-SYN due to FKBP12 was significant from a concentration of 1 μM FKBP12 onwards, as can be seen by a reduction in halftime of about 50% at 1 μM FKBP12 which increased to 74% at 10 μM FKBP12 (Figure 4A). No clear effect of FKBP12 on the mean end phase amplitude was observed (data not shown).

In the case of pS129-α-SYN the enhanced acceleration in the presence of FKBP12, was similar to that of WT α-SYN, with a reduction in halftime of 44% at 1 μM FKBP12 which increased to 69% in the 10 μM FKBP12 condition. Here, a tendency towards reduced halftimes was also seen at lower concentrations of FKBP12 (100 pM) (Figure 4B). Due to a high variation between different measurements no significant differences were observed in the mean end phase amplitudes.

The acceleration due to FKBP12 on pY125-α-SYN was comparable to the acceleration on WT α-SYN, although at the concentration of 100 pM FKBP12 a significantly reduced halftime of about 34% was reached, which increased to 44% in the condition with 1 μM FKBP12 (Figure 4C). As was the case for WT and pS129 α-SYN, here again, no clear effect of FKBP12 on the end phase amplitude was observed (data not shown).

### 2.7. Discussion

The fact that 90% of the fibrillar α-SYN in LBs is phosphorylated on S129 [12–14] raises questions on the role of this phosphorylation event in PD. Since 2005, when the results from Chen and Feany showed a clear correlation between phosphorylation and toxicity in a Drosophila model of PD, increasing research efforts have focused on the phosphorylation events of α-SYN and disease propagation [16]. Phospho-mimicking mutants and the corresponding non-phosphorylatable mutants are now widely used as respectively positive and negative controls for phosphorylated α-SYN both *in vivo* and in cell culture models [16–19,31,41,42]. In the present work, we performed an extensive *in vitro* study of the following phosphorylation mutants for the two most important phosphorylation sites in α-SYN: S129D/A and Y125E/F and compared their behaviour with *in vitro* phosphorylated α-SYN.

#### 2.7.1. The Y125 Phosphorylation Mutants Display Different Aggregation Properties Compared to *in Vitro* Phosphorylated α-SYN

We demonstrate here that the S129D and the S129A mutation do not significantly influence the aggregation properties of α-SYN (although there is a tendency towards faster aggregation for the S129A variant). In contrast, both the Y125E and the Y125F mutations induced a twofold deceleration in the aggregation kinetics compared to wild type. These findings show that phosphorylation mutants at Y125 influence the aggregation process of α-SYN; however the lack of effect in the Y125 phosphorylated form of α-SYN argues against the use of Y125E and Y125F as positive and negative controls in phosphorylation studies since especially the negative control (Y125F) follows different aggregation kinetics than WT α-SYN. These position-related effects raise the question whether the phospho-mimicking mutants reproduce the properties of phosphorylated α-SYN. Paleologou and colleagues performed a similar study on the phosphorylation mutants of S129. They saw a significantly accelerated fibrillization of the S129A mutant compared to WT, while the phospho-mimicking mutant S129D slowed down this process [15]. We observed a similar trend in our assay; however differences did not reach statistical significance. The effects observed for phospho-dead mutants (S129A and Y125F) on aggregation kinetics of α-SYN suggest that structural effects as opposed to charge effects on the α-SYN protein at these positions are contributing to changes in aggregation properties of α-SYN.

Our data stress the importance of single amino acid changes in the aggregation kinetics of α-SYN. This conclusion is in accordance with previous studies reporting that monomeric α-SYN is stabilized by long-range intramolecular interactions between the *C*-terminal portion (res 120–140) and the central NAC domain [3,4,43]. This study proposed that if these interactions are mainly hydrophobic, then the three tyrosines (Tyr) in the *C*-terminal part would be important (Y125, Y133, Y136). Indeed, mutation of these three tyrosine residues to alanines completely inhibited the fibrillization of α-SYN. For the single Y125F mutant only a mild inhibitory effect was observed, which is in agreement with our results. However, contradictory results have also been observed since disruption of the long-range interactions by increasing the temperature caused accelerated aggregation [3]. The explanation might be that in some cases residual interactions are present, which can form off-pathway oligomers, or decrease intermolecular interactions that lead to fibrils by adopting an auto-inhibitory conformation. In favour of the off-pathway oligomer mechanism of action, it has been shown that Tyr nitration stabilizes specific oligomers, which also inhibits fibrillization [44,45].

#### 2.7.2 *. In Vitro* Phosphorylation of α-SYN on S129 or Y125 Does not Affect the Fibrillization Process

Extensive *in vitro* studies have focused on the effect of pS129 on the aggregation properties of α-SYN in order to solve the question at what point this phosphorylation event takes place in LB pathogenesis. As was stated above, promotion as well as inhibition of fibril formation [13,15,22,38] has been observed.

Our data show that pS129-α-SYN displays comparable fibrillization kinetics to the WT protein *in vitro*. Of course, it is interesting to look into the differences in experimental setup between these studies to identify important factors that determine the aggregation kinetics. A first discrepancy between the reported data is the phosphorylation degree of the α-SYN protein used in the fibrillization assays. A second difference is the identity and specificity of the kinase used e.g., CKI (partial phosphorylation, rather aspecific), CKII (partial phosphorylation, rather specific) and PLK2 (strong phosphorylation, specific [26] and our results). For instance, inhibition of α-SYN aggregation has been reported for CKI phosphorylated α-SYN [15], however, this study uses an S87A α-SYN variant (rather than WT) to avoid CKI phosphorylation of S87. Another study examining the role of the S87 site shows that this site can regulate α-SYN fibrillization [38], suggesting that effects of phosphorylation of S129 are different for WT compared to S87A α-SYN, and providing a reconciling explanation for the apparent discrepancy with the present study. Since we only obtained a phosphorylation degree of 44% with PLK2, we cannot rule out the possibility that fully phosphorylated α-SYN aggregates differently. However, in a cellular environment a pool of 100% phosphorylated α-SYN is unlikely. From our data it can be concluded that phosphorylation of α-SYN is not necessary and does not appear to promote the aggregation. This suggests also that phosphorylation of α-SYN affects its interaction with cellular components such as other proteins or metals, as previously has been reported [46].

We could not observe an effect of α-SYN phosphorylation at Y125 on fibrillization kinetics, in contrast with the phospho-mimic mutant Y125E. This strongly suggests that the latter cannot be considered as a positive control for phosphorylation. The phosphorylation degree at Y125 of these samples used in ThioT experiments was determined to be 71%, although Y133 or Y136 were also partially phosphorylated. We cannot fully exclude that our observations may be secondary to effects of phosphorylation at these sites, however the Y125 remains the primary phosphorylated tyrosine in our tests. Our observations are in contrast with previously reported results, where phosphorylation by SYK kinase on all three tyrosines in the *C*-terminus inhibited eosin-induced oligomerisation [27]. This discrepancy might be due to different assays used. Negro *et al.* [27] used a very short incubation period and focused on early oligomer formation while we looked at fibril formation. Moreover, the construct used was a His-tagged form of α-SYN. We have recently shown that the His-tag affects the aggregation behaviour of α-SYN [40]. In contrast, a more recent study using a semi-synthetic α-SYN protein phosphorylated at Y125 shows that aggregation of pY125 α-SYN is comparable to WT α-SYN after 24 and 48 h of aggregation [47]. Crucially, we show in detailed aggregation kinetics experiments that both WT and pY125 α-SYN aggregate at comparable speeds and that half-times of aggregation are less than 24 h, showing that measures at short time points (24 h or less) are required to observe differences using a ThioT readout.

Research on Y125 phosphorylation has been primarily focused on the identification of possible kinases and implications for the normal function of α-SYN. pY125-α-SYN has only recently been linked with the pathogenesis of PD. Chen and Feany showed that increased phosphorylation on Y125 decreased neurotoxicity in a Drosophila model of PD [16]. Interestingly this correlated with less oligomer formation. pY125-α-SYN could even rescue the toxicity caused by overexpression of the ‘phospho-mimicking’ S129D mutant [31]. Moreover, a triple tyrosine mutant Y(125,133,136)A caused accelerated neurotoxicity and enhanced oligomer formation, whereas it completely inhibited fibrillization *in vitro*, possibly by formation of stable off*-*pathway oligomers as mentioned before [48].

In humans as well as in transgenic Drosophila, phosphorylation on Y125 decreases with age. In patients with Dementia with Lewy Bodies no Y125 phosphorylation could be detected in contrast to the controls [31]. This might mean that one of the first necessary steps in the pathogenesis of PD is loss of this protective pY125-α-SYN. Our own data show that pY125-α-SYN still has the capacity to form fibrils at rates comparable to WT, but in a cellular environment this might be prevented through interactions with other proteins.

#### 2.7.3. FKBP12 Mediated Acceleration of Fibrillization Is Unaltered for Phosphorylated α-SYN

FKBP12 is a FK506 binding protein belonging to the family of immunophilins. These enzymes all have *cis-trans* peptidyl prolyl isomerase (PPIAse) activity [49]. It has been shown that FK506, an immunosuppressive drug, and related non-immunosuppressive analogs, exhibit neuroregenerative and neuroprotective properties [50–52]. Our group demonstrated an accelerating effect of FKBP12 on the aggregation of α-SYN *in vitro* [33,39], an effect that is attributed to FKBP12’s PPIase activity as an enzymatically inactive FKBP12 does not influence α-SYN fibril formation [33]. More recently involvement of FKBPs in a cell culture model of synucleinopathy was reported [34]. Moreover, in mouse brain FK506 also reduced α-SYN aggregation and neurodegeneration [34]. In this study we addressed the question whether phosphorylation on S129 and/or Y125 could affect the accelerating ability of FKBP12 on the aggregation of α-SYN *in vitro*. Indeed, both residues are in close proximity of proline residues (P108, P117, P120, P128, and P138) which are the likely targets of FKBP12 action [40]. Our data show that FKBP12 can enhance the fibrillization of phosphorylated α-SYN comparable to the observed acceleration seen with WT α-SYN. However, we cannot exclude that phosphorylation might still play a regulatory role in a cellular environment.

## 3. Experimental Section

### 3.1. Purification of α-Synuclein (α-SYN)

Wild type (WT) α-SYN (140 aa) and its mutants were expressed and purified as described before [53].

### 3.2. Construction of Phosphorylation Mutants of α-Synuclein (α-SYN)

The following mutations were made using site-directed mutagenesis (QuikChange XL, Stratagene, La Jolla, CA, USA): S129A-α-SYN, S129D-α-SYN, Y125F-α-SYN and Y125E-α-SYN, according to the manufacturer’s protocol. For fluorescent labeling of α-SYN, the A140C mutant gene was kindly provided by Prof. V. Subramaniam (Biophysical Engineering Group, Unversity of Twente, Enschede, The Netherlands). The purification protocol used for the phosphorylation mutants was identical to that of WT protein.

### 3.3. SDS PAGE

Ten microgram of WT α-SYN or its mutants was loaded on a 16% polyacrylamide Sodium Dodecyl Sulphate gel after addition of SDS loading dye, boiling for five min and a short centrifugation step of each sample. Gels were run at 35 mA for 1 h in an electrophoresis tray.

### 3.4. Fluorescence Correlation Spectroscopy (FCS)

In fluorescence correlation spectroscopy (FCS), the diffusion coefficient of a fluorescent molecule is calculated from the autocorrelation function of the fluctuating fluorescence signal, resulting from the diffusion of fluorescent molecules through the confocal volume.

Measurements were performed on the LSM 510/ConfoCor II combination (Zeiss, Jena, Germany). Experiments were done with 5–6 nM labeled protein [A140C-Alexa 488-α-SYN, (Alexa 488–C_5_-maleimide (Molecular Probes, Life Technologies, Carlsbad, CA, USA))] mixed with different concentrations of unlabeled protein (30–100 μM), to ensure that the number of fluorescent molecules in the confocal volume (0.312 fL) is limited to 1–10. For mathematical background and labeling procedure see Supporting Information.

### 3.5. Circular Dichroism

α-SYN WT and mutants were centrifuged for 30 min at 8500 *g* (Galaxy 14D centrifuge, Sorvall, VWR, Geel, Belgium) prior to the experiments to remove pre-existing aggregates. Samples were incubated at 37 °C while stirring (~200 rpm). Far-UV CD spectra were recorded on a Jasco-810 spectrophotometer (Jasco, IJselstein, The Netherlands) in a 1 mm path length Quartz cuvette at room temperature. Spectra were recorded from 195 to 260 nm with a step size of 1 nm and a scanning speed of 20 nm/min. An average of 3 scans was recorded. All spectra were corrected by subtracting the background spectrum of the buffer. For every time point, aliquots were taken from the samples and diluted to 0.2 mg/mL in 20 mM Tris-HCl, pH 7.4, at 20 °C.

### 3.6. Turbidity and Thioflavin T Measurements

Prior to each experiment, the pre-existing aggregates were removed by centrifugation as described above. The samples were further diluted in 20 mM Tris-HCl pH 7.4 to the indicated concentrations and NaCl was added up to 100 mM, unless otherwise stated. For thioflavin T experiments 50 μM of ThioT (Sigma-Aldrich, St. Louis, MO, USA) was added.

All samples were incubated in a flat-bottom transparent 96-well plate (Greiner, Bio-One, Hemmel, Belgium) at 37 °C with continuous shaking (~270 rpm) in a Safire^2^ plate reader (TECAN, Mechelen, Belgium). Every 1000 s, the turbidity of the samples was determined by measuring the absorbance at 350 nm, ThioT fluorescence was measured at 482 nm (excitation at 446 nm). To reduce the number of data points and simplify the graphics, every fifth time point was visualized. The samples were made in triplicate and each experiment was repeated at least three times independently. Every graph is a representative example.

### 3.7. Data Fitting and Statistical Analysis of ThioT and Turbidity Data

All aggregation curves were fitted with SIGMAPLOT version 8 (Systat Software Inc., San Jose, CA, USA) to a three-parameter sigmoidal model to extract the relevant aggregation parameters (see Equation (1)).

(1) $$y=\frac{a}{1+e^{-(x-x_{0}+b)}}$$

in which *y* is the turbidity or ThioT fluorescence signal, *x* is the time, *a* is the total increase in absorbance or fluorescence, *x*_0_ is the half-time of aggregation and 1/*b* describes the slope of the curve at its mid-point and is also the rate constant of aggregation at this point. For statistical analysis all groups were first compared to identify significant differences through an ANOVA one-way analysis of variance, followed by a Tukey’s post test to compare all pairs of columns.

### 3.8. Phosphorylation Assay

Two microgram of α-SYN was incubated with the respective kinases, 6 μCi of ATP-γ-phosphate (PerkinElmer, Norwalk, CT, USA) and 10 μM of cold ATP in buffer A (20 mM Tris pH 7.4, 10 mM MgCl_2_, 5 mM β-glycerophosphate, 0.1 mM Na_3_VO_4_, 2 mM DTT) in a total volume of 10 μL for the indicated time points. The reaction was stopped by adding 1% SDS solution and boiling for 5 min.

The following concentrations of the kinases were used: 7.9 nM/reaction of Casein Kinase II (CKII) (Specific activity = 2540 pmol/μg·min) (Biaffin, Kassel, Germany); 320 nM/reaction of Polo-like kinase 2 (PLK2) (Specific activity = 69 pmol/μg·min) (Proqinase, Freiburg, Germany); 164 nM/reaction of Sarcoma (SRC) kinase (Specific activity = 142 pmol/μg·min) (Biaffin); 103 nM/reaction of FYN kinase (Specific activity = 87 pmol/μg·min) (Proqinase).

Samples were resolved on a NuPAGE 10% bis-Tris pre-casted gel (Invitrogen, Merelbeke, Belgium) in 1× MES buffer. The samples were transferred to a nitrocellulose membrane by semi-dry blotting according to the manufacturer’s protocol. Phosphate incorporation was measured by autoradiography where the membrane was exposed to a phosphorescence plate (GE healthcare, Sunnyvale, CA, USA). The densitometric quantification of the autoradiogram was done using AIDA image analyzer (Software Verise, Straubenhardt, Germany). Equal loading was confirmed by western blotting using an anti-α-SYN antibody (see below).

The degree of phosphorylation of alpha-synuclein was also tested for these samples in a separate experiment, in which the radioactively phosphorylated α-SYN was separated via SDS-PAGE (as described above) and the gel stained using Coomassie Brilliant Blue according to the manufacturer’s instructions (Thermo Scientific, Pittsburgh, PA, USA). Gel bands were excised and submitted to scintillation counting and calculation of incorporated phosphates, as previously described [54].

In order to prepare *in vitro* phosphorylated α-SYN for further experiments (including far UV-CD measurements, mass spectrometry analysis as well as fibrillization experiments), the following protocol was used: 1 mg of recombinant α-SYN was phosphorylated with CKII, PLK2, SRC or Fyn kinase in 600 μL buffer A. Each reaction was incubated overnight at 30 °C in the presence of 1 mM ATP. The concentration of kinase used was 61.7 μM for CKII, 17.22 μM for Fyn kinase, 5.42 μM for PLK2 and 27.3 μM for SRC kinase; the concentration of α-SYN was 115 μM. After phosphorylation and prior to the aggregation assay pre-existing aggregates were removed by centrifugation at 8500 *g* for 30 min. Since ATP itself had an effect on the aggregation of α-SYN (see Supplementary Figure S3), it was removed by loading each sample on a PD-10 column. Elution was done in 100 μL fractions with 20 mM Tris pH 7.4. For the aggregation assays 30 μM of phosphorylated α-SYN in buffer B (20 mM Tris-HCl pH 7.4, 100 mM NaCl) was used unless otherwise stated. The control was subjected to the same conditions without addition of ATP and kinase. Protein concentrations were re-determined after the PD-10 buffer exchange step prior to further testing.

### 3.9. Mass Spectrometry

Phosphorylated α-SYN (50 μg) was subjected to a TCA-acetone precipitation and digested for 3 h at 50 °C with 12 μg of thermolysin in the presence of 200 mM ammonium acetate and 20 mM CaCl_2_. For more information about the actual procedure see Supporting Information.

### 3.10. Western Blot Analysis

After SDS-PAGE, proteins were blotted on a PVDF membrane (Bio-Rad Laboratories, Hercules, CA, USA), which was rehydrated in methanol (2 s) and PBS (2 × 5 min) before use. After blotting (30 V, 1 h), the membrane was blocked in PBS-0.1% Triton (PBST) with 5% milk (Régilait skim milk, Saint-Martin-Belle-Roche, France) to avoid unspecific binding of the antibodies used. This was followed by incubation with the monoclonal mouse anti-α-SYN primary antibody (Zymed, San Francisco, CA, USA, 1:500 dilution in PBST-5% milk, 1 h), by a wash step (4 × 10 min, PBST), a secondary antibody incubation (horse radish peroxidase (HRP)-labeled goat anti-mouse polyclonal antibody (DAKO, Glostrup, Denmark), 1:10,000 dilution in PBST, 30 min) and a final wash step (4 × 10 min, PBST). Protein bands were visualized with the ECL detection kit (GE healthcare) using a LAS-3000 Mini imager (Fujifilm, Tokyo, Japan). Blots were analysed by the AIDA image analyzer software.

### 3.11. Transmission Electron Microscopy (TEM)

For the TEM measurements, 300-mesh carbon and formvar coated copper grids were used. After adsorption (for 2 min) of the α-SYN samples onto the grids, they were negatively stained with 1% uranyl acetate for 30 s. The samples were examined with a Zeiss EM 10C electron microscope JEOL JEM2100 LaB6 (Zeiss, Zaventem, Belgium) operating at 200 KeV. A magnification of 5000 was used to get an overview image. For more detailed pictures of the fibrils a magnification of 20,000 was used.

### 3.12. Peptidyl Prolyl *Cis-Trans* Isomerase Activity of FKBP12

For more information about the activity assay see Supporting Information.

## 4. Conclusions

In normal physiological conditions only about 4% of α-SYN is phosphorylated on S129 [14,17]. In this study we show that phosphorylation on S129 is not necessary for, or does not appear to promote, the aggregation of α-SYN, since its fibrillization kinetics are comparable to that of the WT protein. Previous work however shows that an increase in pS129-α-SYN is observed under certain conditions such as proteasomal inhibition, oxidative stress, mitochondrial complex 1 dysfunction and an increase in iron levels, which are all implicated in PD [17,55,56]. This elevated pS129 could be caused by upregulated kinase activity, as has been shown for CKII [22,56], by decreased phosphatase activity or a decrease in degradation of phosphorylated α-SYN. For these reasons, the reversal of upregulated pS129 by pharmacological inhibition of kinases phosphorylating S129 has been proposed as a therapeutic venue for PD [57]. Given our data that phosphorylation mutants behave so differently biochemically from phosphorylated α-SYN, it may be interesting to develop cellular and *in vivo* disease models based on the modulation of pS129 levels via manipulation of the kinases and phosphatases regulating that phospho-site. Our study also indicates that future research is warranted to study the influence of FKBPs on cellular aggregation of phosphorylated α-SYN as well as the study of other effectors of α-SYN aggregation and their effect on the aggregation kinetics of the phosphorylated protein.

## Supporting Information

### Fluorescent Labeling

For fluorescent labeling of α-SYN the A140C mutant is used, which was purified as described above. The protein (200 μM) was labeled with a 20-fold molar excess of Alexa 488–C_5_-maleimide (Invitrogen, Carlsbad, CA, USA) in 20 mM Tris buffer pH 7.4. The protein was incubated for 2 h at room temperature while stirring (100 rpm). Labeled protein was separated from free dye by gel chromatography using a double PD-10 column (GE Healthcare). *C*-terminal Alexa labeled α-SYN was used for FCS measurements. The efficiency of labeling is approximately 42% and was calculated using the extinction coefficients ɛ_(280 nm (α-SYN))_ = 5800, ɛ_(280 nm (Alexa Fluor 488 nm))_ = 920 and ɛ_(493 nm (Alexa Fluor 488 nm))_ = 72,000 cm^−1^ M^−1^ through use of the following equation:

(S1) $$A_{280nm(protein)}=A_{280nm(protein)}-A_{493nm(Alex488)}\frac{{ɛ}_{280nm(Alexa488)}}{{ɛ}_{493nm(Alexa488)}}$$

The aggregation rate of A140C is comparable to WT as measured by turbidity and thioflavin T (ThioT) fluorescence [58].

### Fluorescence Correlation Spectroscopy (FCS)

A small fraction (<10%) of free dye is present in all measured samples, probably due to the presence of non-specifically bound dye molecules that are not fully removed by the gel chromatography after labeling, and are released upon dilution to nanomolar concentrations. For each time point, 16 measurements were done, each with a time lapse of 10 s, and every curve was fitted using the following two component model (Equations (S2–S4)):

(S2) $$G(\tau )=1+G_{T}(\tau )\times G_{D}(\tau )$$

(S3) $$G_{T}(\tau )=\left(1+\frac{T_{R}e^{-\tau /\tau_{x}}}{1-T_{R}}\right)$$

(S4) $$G_{D}(\tau )=\left(\frac{1}{N}\right)\left\{\left(\frac{F_{1}}{1+\tau /\tau_{1}}\right){\left(\frac{1}{1+{\left(\omega_{x}/\omega_{z}\right)}^{2}(\tau /\tau_{1})}\right)}^{1/2}+\left(\frac{1-F_{1}}{1+\tau /\tau_{2}}\right){\left(\frac{1}{1+{\left(\omega_{x}/\omega_{z}\right)}^{2}(\tau /\tau_{2})}\right)}^{1/2}\right\}$$

*G*_T_ is the part of the autocorrelation curve at a fast timescale, representing the photodynamics; *G*_D_ is the concentration-dependent part representing diffusion; is the correlation time; and are the amplitude and the relaxation time of the photodynamic process; *N* is the average number of particles in the confocal volume; (*F*_1_) and (1 − *F*_1_) are respectively the diffusion time (fraction) of free Alexa dye and dye bound to α-SYN; and are the radial and axial radii of the confocal volume, which are determined in the calibration with Alexa 488 (*D* = 435 μm^2^ s^−1^) [59] and are fixed throughout the measurements.

### Mass Spectrometry

Identification of phosphopeptides in thermolysin digested α-synuclein after SNK and FYN phosphorylation was executed both with MALDI-TOF/TOF and LC-ESI-MS/MS. In brief, one tenth of the sample of phosphorylated α-synuclein (note: the same sample which is used in the *in vitro* aggregation tests) was subjected to phospho-enrichment on PhosTrap beads (Perkin Elmer, Waltham, MA, USA) according to manufacturer’s instructions. The PhosTrap eluate was divided in two for MALDI and ESI analysis, respectively. These eluates were used to detect the nature of the phosphorylated species in the thermolysin digest. The identity of the peptides was confirmed by MS/MS in both ionization modes. In the next step, the non-phosphorylated counterparts of these phosphopeptides were quantified in non-phosphoenriched samples. MALDI analysis was executed on a 4800 MALDI TOF/TOF instrument (ABSCIEX, Framingham, MA, USA) in the reflectron mode. ESI analysis was executed on a 4000 QTRAP instrument (ABSCIEX) coupled to a Dionex Ultimate capillary liquid chromatography system. Peptides were separated on a PepMap C18 column developed with a 30 min linear gradient (0.1% formic acid-6% acetonitrile-water to 0.1% formic acid–40% acetonitrile–water). MRM (multiple reaction monitoring)-induced product (+) ion scanning was used to identify and quantify (MultiQuant 1.1, ABSCIEX, Framingham, MA, USA) peptides.

Calculation of percentage phosphate incorporation, was executed based on the quantification of non-phosphorylated equivalents of the phosphopeptides in non-phosphorylated synuclein, SNK phosphorylated synuclein and FYN phosphorylated synuclein, adapted from a method described by Olsen and colleagues [60] (Olsen *et al*. 2010), which was originally developed to calculate peptide phosphorylation stoechimetries if MS data of phosphopeptides (P), of the corresponding non-phosphopeptides (NP), and of relative protein abundance (*z*) are available in two conditions (H and L) of a SILAC experiment.

(S5) $${\text{P}}_{\text{H}}+{\text{NP}}_{\text{H}}=z\;({\text{P}}_{\text{L}}+{\text{NP}}_{\text{L}})$$

(S6) $${\text{P}}_{\text{H}}/{\text{P}}_{\text{L}}=x$$

(S7) $${\text{NP}}_{\text{H}}/{\text{NP}}_{\text{L}}=y$$

(S8) $$\text{A a result}:{\text{P}}_{\text{L}}/{\text{NP}}_{\text{L}}=(z-y)/(x-z)$$

In the adapted calculation employed here, the H and L conditions are replaced with CTRL and SNK (or FYN), respectively. In this way, the following equations are obtained:

(S9) $${\text{NP}}_{\text{CTRL}}/{\text{NP}}_{\text{SNK}}=y$$

(S10) $${\text{P}}_{\text{SNK}}/{\text{NP}}_{\text{SNK}}=(z-y)/(0-z)$$

*x* = 0 because there is no phosphorylation in CTRL condition;

*y* is known from relative abundancies of non-phosphopeptides in MS;

*z* is known from initial protein concentration measurements.

### Peptidyl prolyl *Cis-Trans* Isomerase Activity of FKBP12

The isomerase activity of FKBP12 was determined following the protocol of Kullertz *et al* [61] (Kullertz *et al*. 1998). Four microliters of FKBP12 or 4 μL of buffer (35 mM Hepes pH = 7.4) was added to 40 μL chymotrypsin (1 g/L in 35 mM Hepes pH = 7.4). After addition of the substrate (400 μL of succinimidyl-Phe-Pro-Phe-4-nitroanilide (Bachem, Torrance, CA, USA)) the reaction was monitored at 390 nm. The obtained curves were fitted to the equation below (Equation (S11)) to obtain the first order rate constant of the reaction.

(S11) $$\text{Y}=A_{0}\;\left(1-e^{-k_{obs}t}\right)+B_{0}$$

The specific enzymatic activity was determined using Equation (S12):

(S12) $$\frac{k_{cat}}{K_{M}}=\frac{k_{obs}-k_{0}}{\left[enz\right]}$$

[*enz*] = concentration of hFKBP12 used, *k*_0_ = the observed rate constant of the uncatalyzed reaction and *k*^obs^ = the observed rate constant in the presence of FKBP12. *K*^M^ is the Michaelis-Menten constant and *k*^cat^ = the catalytic rate constant.

Figure S1. General aggregation properties of WT α-SYN and phosphorylation mutants. The aggregation kinetics were monitored by turbidity measurements at 350 nm under continuous shaking (270 rpm) at 37 °C. The concentration of α-SYN was 50 μM. (**A**) Representative measurement showing the same trends as observed in ThioT experiments, both tyrosine phosphorylation mutants aggregate slower (Y125F (white squares) and Y125E (gray squares)) while both serine phosphorylation mutants (S129A (white triangles), S129D (gray triangles) aggregate as fast as WT α-SYN (white circles); (**B**) Mean halftimes of at least three independent measurements (each done in triplicate) with SEM shown on each bar; and (**C**) Mean end phase amplitude. * indicates a statistical significant difference when compared to WT with a p-value < 0.05.

Figure S2. Size distribution of (phosphorylated)-α-SYN before aggregation using FCS. Measure of diffusion coefficients of phosphorylated α-SYN compared to the non-phosphorylated control using FCS. pS129-α-SYN (dark yellow): phosphorylated α-SYN on S129 using PLK2, pY125-α-SYN (dark blue): phosphorylated α-SYN using Fyn kinase. In order to perform FCS, trace amounts of A140C-α-SYN, labeled with Alexa 488 nm was added, as described in the Materials and Methods section (**A**) Mean D of at least four independent measurements, SEM shown on each bar. No significant difference was seen between the mean D (86.4 +/− 4.95 to 92.3 +/− 3.8 μm^2^/s), corresponding to the monomeric form of the protein; and (**B**) Mean hydrodynamic radius calculated from D assuming a spherical particle. No significant difference was observed between p129-, pY125- and WT ctl-SYN.

Figure S3. Effect of ATP on the aggregation kinetics of a-SYN. The kinetics of the fibrillization process of α-SYN WT in the presence and absence of ATP followed by a Thioflavin T assay under continuous shaking (270 rpm) at 37 °C. A concentration of 50 μM α-SYN was used in each experiment. (**A**) Representative figure showing an inhibitory effect of ATP in α-syn fibrillization. The halftimes of fibrillization are expressed as percentages of WT, which was set to 100%; (**B**) Mean values of the halftimes; (**C**) Mean end phase fluorescence intensities; (**B**) and (**C**) are calculated from five independent measurements (*n* = 5) each done in quadruplicate, with the standard error of mean (SEM) shown on each bar; and (**D**) Laser scanning microscopy images at the slide surface during the course of the fluorescence correlation spectroscopy (FCS) experiments. Scale bar, 50 μm (valid for all panels). In these experiments, alpha-synuclein is mixed with fluorescently labelled alpha-synuclein as described in materials and methods. Shown here is that the presence of ATP in the analysis mixture leads to rapid deposition of large amorphous aggregates, in contrast to the deposition of aggregates of fibrillar form in normal samples. These experiments illustrate that the presence of ATP in the *in vitro* aggregation assays cause a time dependent non-fibrillar aggregation of alpha-synuclein, explaining the low end phase fluorescence intensity in the ThioT assay. For this reason, ATP is removed from all aggregation assays of this study, as described in Materials and Methods.

Figure S4. ESI-MS/MS spectra of phosphopeptides of thermolysin digested α-synuclein after (**A**) SNK phosphorylation and (**B**) FYN phosphorylation.
