# Supplementary figures and images for: In Vitro Phosphorylation Does not Influence the Aggregation Kinetics of WT α-Synuclein in Contrast to Its Phosphorylation Mutants
Source: Int J Mol Sci. 2014 Jan 15;15(1):1040–67. doi: 10.3390/ijms15011040 (PMC3907855; doi:10.3390/ijms15011040)

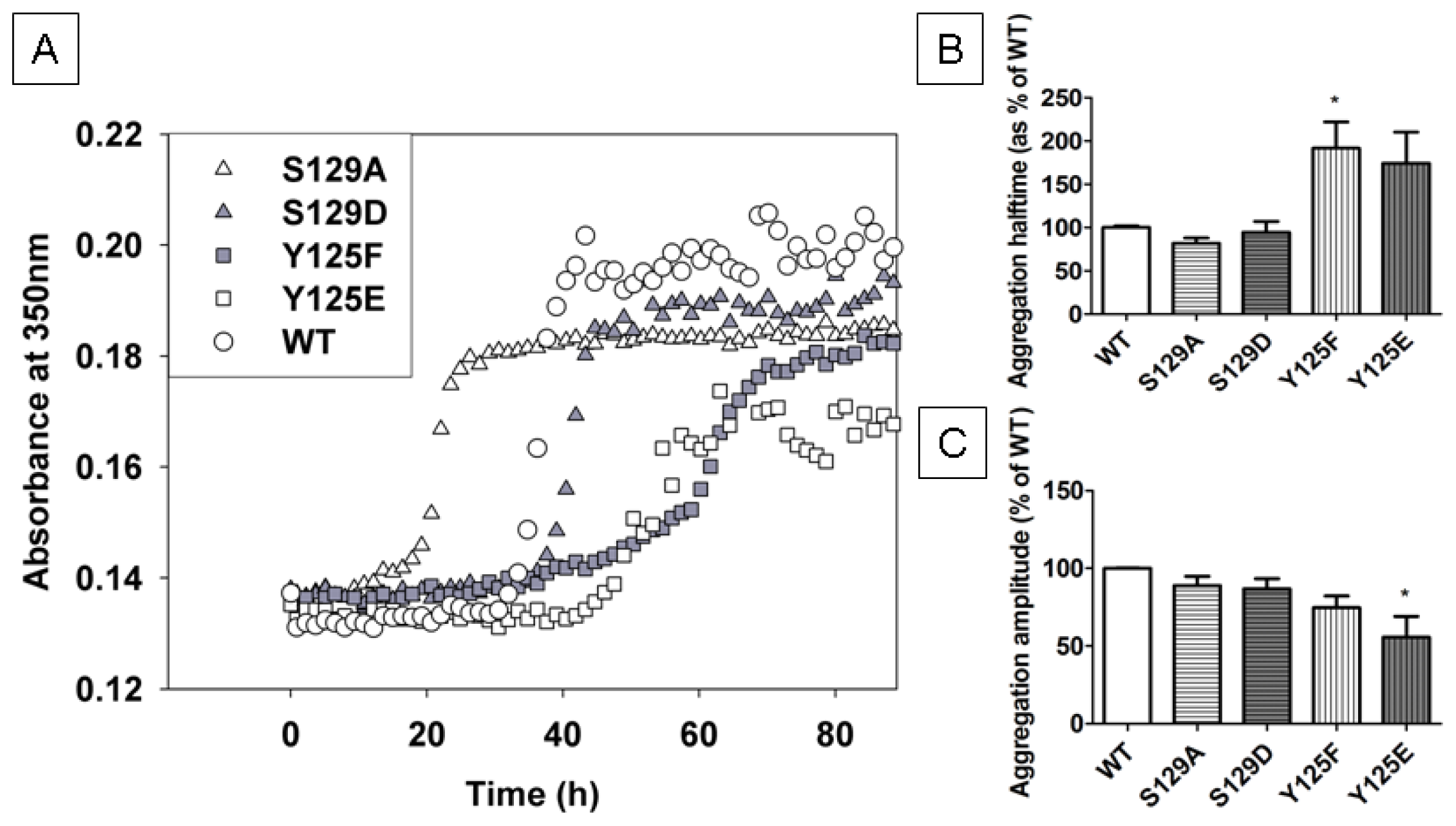

Supplement: Figure S1. — General aggregation properties of WT α-SYN and phosphorylation mutants. The aggregation kinetics were monitored by turbidity measurements at 350 nm under continuous shaking (270 rpm) at 37 °C. The concentration of α-SYN was 50 μM. (A) Representative measurement showing the same trends as observed in ThioT experiments, both tyrosine phosphorylation mutants aggregate slower (Y125F (white squares) and Y125E (gray squares)) while both serine phosphorylation mutants (S129A (white triangles), S129D (gray triangles) aggregate as fast as WT α-SYN (white circles); (B) Mean halftimes of at least three independent measurements (each done in triplicate) with SEM shown on each bar; and (C) Mean end phase amplitude. * indicates a statistical significant difference when compared to WT with a p-value < 0.05. [file ijms-15-01040s1.tif]

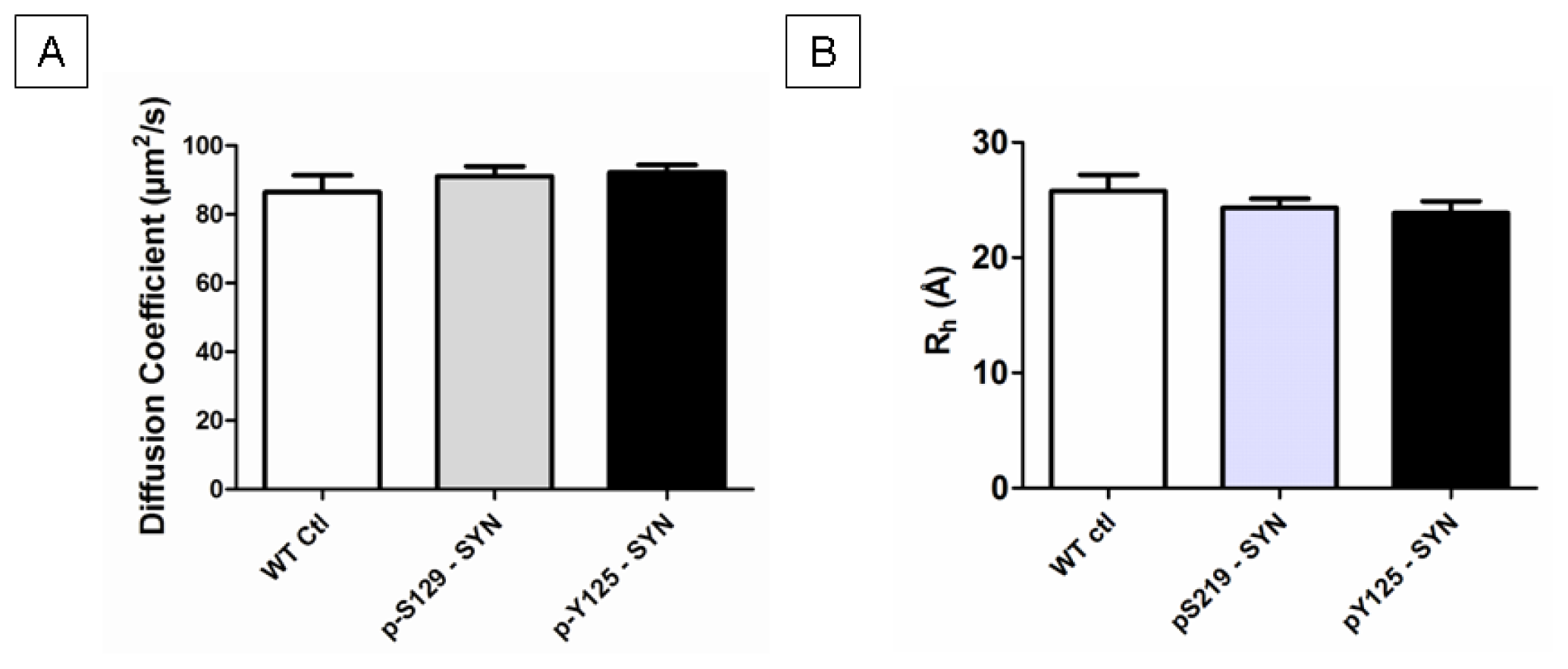

Supplement: Figure S2. — Size distribution of (phosphorylated)-α-SYN before aggregation using FCS. Measure of diffusion coefficients of phosphorylated α-SYN compared to the non-phosphorylated control using FCS. pS129-α-SYN (dark yellow): phosphorylated α-SYN on S129 using PLK2, pY125-α-SYN (dark blue): phosphorylated α-SYN using Fyn kinase. In order to perform FCS, trace amounts of A140C-α-SYN, labeled with Alexa 488 nm was added, as described in the Materials and Methods section (A) Mean D of at least four independent measurements, SEM shown on each bar. No significant difference was seen between the mean D (86.4 +/− 4.95 to 92.3 +/− 3.8 μm2/s), corresponding to the monomeric form of the protein; and (B) Mean hydrodynamic radius calculated from D assuming a spherical particle. No significant difference was observed between p129-, pY125- and WT ctl-SYN. [file ijms-15-01040s2.tif]

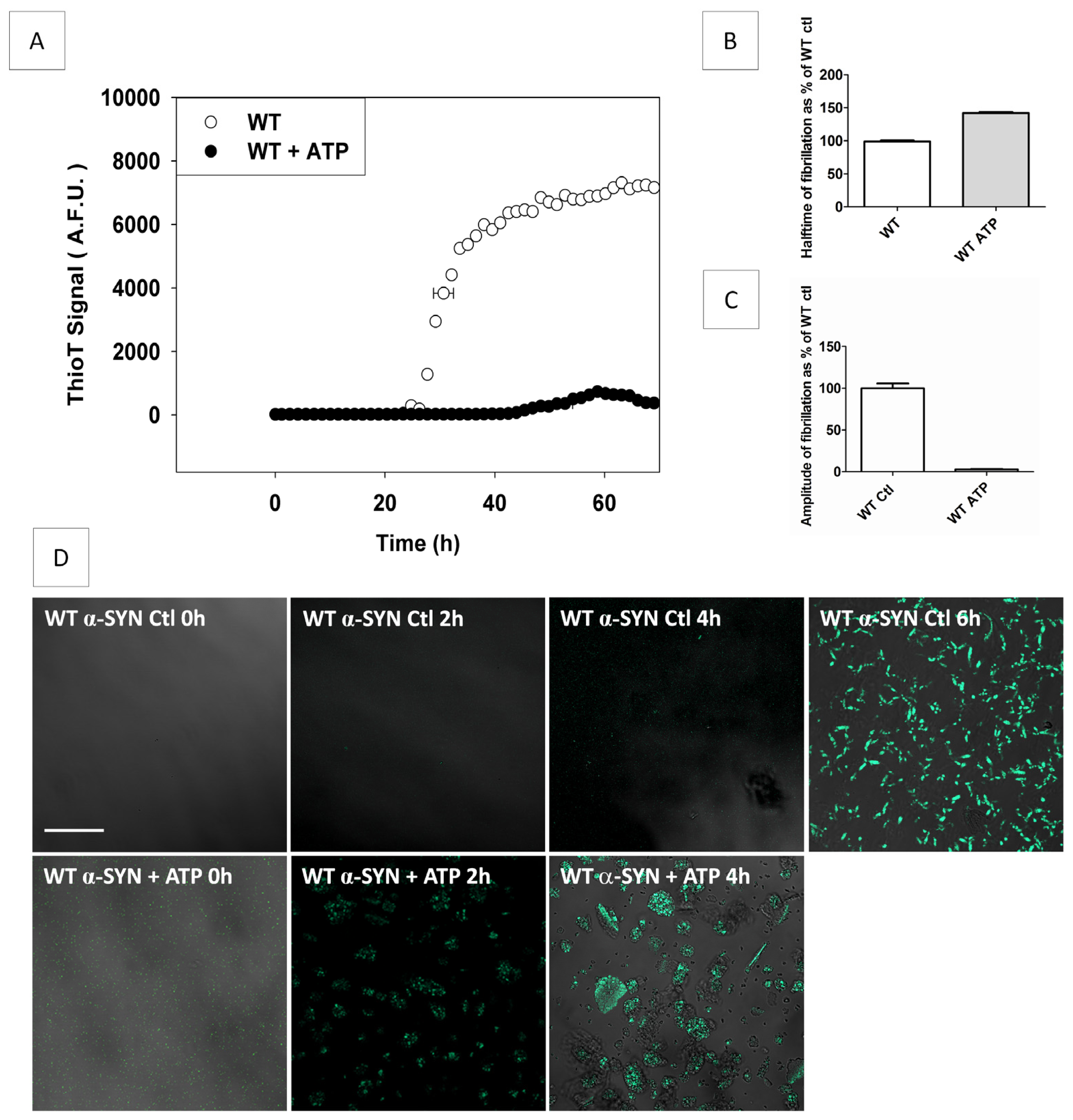

Supplement: Figure S3. — Effect of ATP on the aggregation kinetics of a-SYN. The kinetics of the fibrillization process of α-SYN WT in the presence and absence of ATP followed by a Thioflavin T assay under continuous shaking (270 rpm) at 37 °C. A concentration of 50 μM α-SYN was used in each experiment. (A) Representative figure showing an inhibitory effect of ATP in α-syn fibrillization. The halftimes of fibrillization are expressed as percentages of WT, which was set to 100%; (B) Mean values of the halftimes; (C) Mean end phase fluorescence intensities; (B) and (C) are calculated from five independent measurements (n = 5) each done in quadruplicate, with the standard error of mean (SEM) shown on each bar; and (D) Laser scanning microscopy images at the slide surface during the course of the fluorescence correlation spectroscopy (FCS) experiments. Scale bar, 50 μm (valid for all panels). In these experiments, alpha-synuclein is mixed with fluorescently labelled alpha-synuclein as described in materials and methods. Shown here is that the presence of ATP in the analysis mixture leads to rapid deposition of large amorphous aggregates, in contrast to the deposition of aggregates of fibrillar form in normal samples. These experiments illustrate that the presence of ATP in the in vitro aggregation assays cause a time dependent non-fibrillar aggregation of alpha-synuclein, explaining the low end phase fluorescence intensity in the ThioT assay. For this reason, ATP is removed from all aggregation assays of this study, as described in Materials and Methods. [file ijms-15-01040s3.tif]

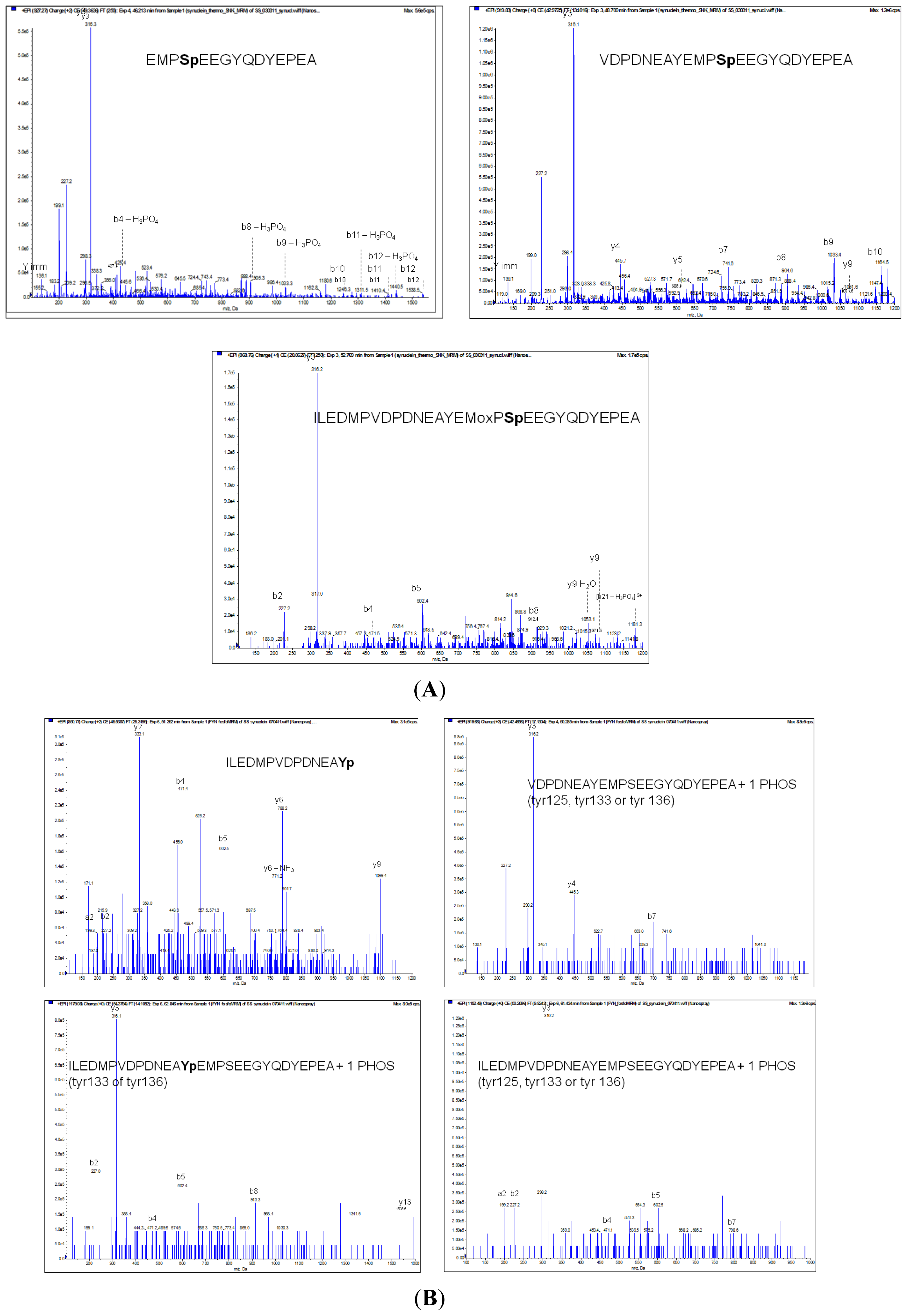

Supplement: Figure S4. — ESI-MS/MS spectra of phosphopeptides of thermolysin digested α-synuclein after (A) SNK phosphorylation and (B) FYN phosphorylation. [file ijms-15-01040s4.tif]
